# Supplementary material for: Antagonization of Ghrelin Suppresses Muscle Protein Deposition by Altering Gut Microbiota and Serum Amino Acid Composition in a Pig Model
Source: Biology (Basel). 2022 May 30;11(6):840. doi: 10.3390/biology11060840 (PMC9220191; doi:10.3390/biology11060840)
Supplement: Supplementary file 1 [file biology-11-00840-s001.zip › biology-1699111-supplementary.pdf]

Supplementary Material

Table S1. Composition and nutrient level of the diet (air-dry basis).

Ingredient	Percentage (%)	Nutritional Compositions (%)	
Corn	70.0	Digestive energy（MJ/kg）	14.60
Soybean meal	18.0	Crude protein	16.00
Wheat bran	6.50	Lysine	1.23
Soybean oil	1.90	Methionine + Cystine	0.70
Lysine	0.69	Threonine	0.79
Methionine	0.24	Tryptophan	0.22
Threonine	0.30		
Tryptophan	0.07		
Calcium hydrogen phosphate	0.45		
Stone powder	0.50		
Salt	0.30		
Multivitamins [1]	0.03		
Minerals [2]	0.20		
Choline chloride (50%)	0.12		
Zeolite powder	0.60		
Antioxidant	0.05		
Antifungal agent	0.05		
Total	100.0		

[1] The mineral supply per kg diet was as follows: Fe 165 mg, Zn 165 mg, Cu 16.5 mg, Mn 30 mg, Co 0.15 mg, I 0.25 mg, and Se 0.25 mg. [2] The multivitamin supply per kg diet was as follows: VA 11,000 IU, VD3 1000 IU, VE 16 IU, VK1 1 mg, VB1 0.6 mg, VB2 0.6 mg, d-pantothenic acid 6 mg, nicotinic acid 10 mg, VB12 0.03 mg, folic acid 0.8 mg, and VB6 1.5 mg.

Table S2 Primers used in this study

Gene	Primer sequence (5'→3')	References	Annealing temp.(°C)
MCT-1	F：TGATGGACCTTGTTGGACCTC<br>R：GAGACGACCTAAAAGTGGTGG	Franziska et al. (2021)	60
PPAR-δ	F：CTCTTCCTCAACGACCAGGT<br>R：GCAGCCCATCCTTATTGACG	Wang et al. (2019)	60
HDAC1	F：ATGAGGAGGGAGAAGGTG<br>R：GGTTGTGGGATAAAGACG	Xu et al. (2021)	60
mTOR	F：AGCCCATAAGAAAACGGGGA<br>R：AAAGGACACCAGCCGATGTA	Tian et al. (2021)	60
S6K1	F：AATACGACAGCCGAACTCCG<br>R：TCACACATCCCCTTCCCACC	Tian et al. (2021)	60
rpS6	F：TGTCCGTCAGTATGTAGTGAGAAAG<br>R：ATTTTTCTTGGTACGTTGTTTCTTC	Jiao et al. (2011)	60
FOXO1	F：AATCGAGTTACGGAGGCATGG<br>R：TAGGGCCCATCAGCACATTC	Du et al. (2021)	60
Beclin-1	F：GATGGTGGCTTTCCTGGACTGTG<br>R：ACTGCCTCCTGTGTCTTCAATCTTG	Wang et al. (2022)	60
LC3-I	F：GCCTTCTTCCTGCTGGTGAACC<br>R：GGGAGGCGTAGACCATGTAGAGG	Wang et al. (2022)	60
LC3-II	F：AAGCCTTCTTCCTGTTAGTGAACGG<br>R：TCCATCTTCATCCTTCTCGCTTTCG	Wang et al. (2022)	60
MYOD	F：TGCAAACGCAAGACCACTAA<br>R：GCTGATTCGGGTTGCTAGAC	Kim et al. (2020)	60
MYOG	F：GGCTACGAGCGGACTGA<br>R：GACACGGACTTCCTCTTACAC	Yang et al. (2015)	60
Atrogin-1	F：CAAAGGCTAAGTGATGGCCG<br>R：GAGGGTAGCATCGCACAAGT	Du et al. (2021)	60
MuRF1	F：GGGCCATCTTCCTCTTGAGT<br>R：GGTGTGCTTCTTCCTTTCCC	Hu et al. (2019)	60

Note: F: forward; R: reverse

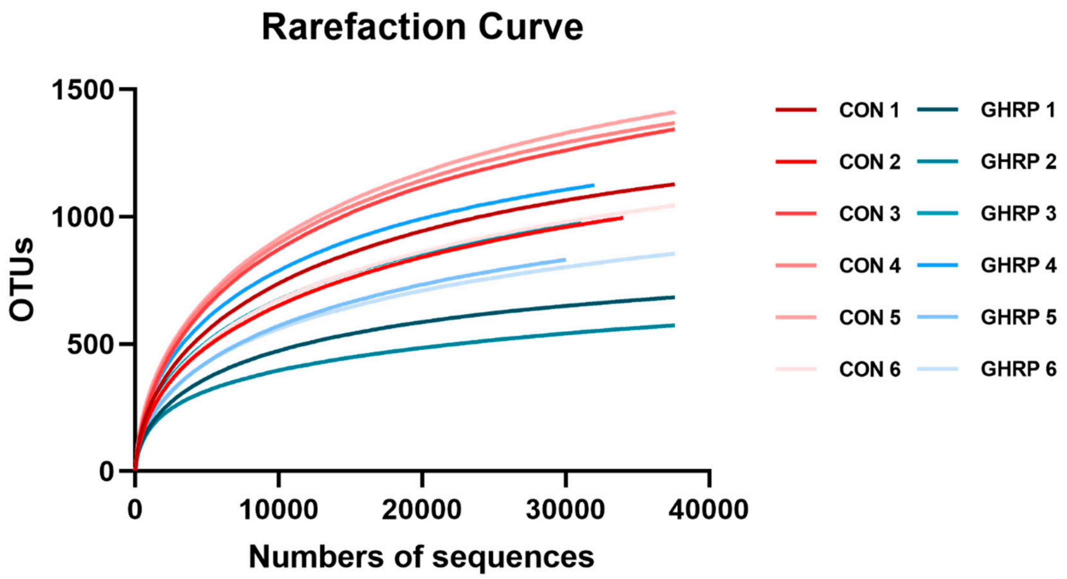

Figure S1. Rarefaction curves comparing the number of sequences with the number of OTUs found in the 16S rRNA gene libraries from microbiota in the colon of pigs in CON and GHRP groups. CON, Injection of saline; GHRP, Injection of [D-Lys3]-GHRP-6.

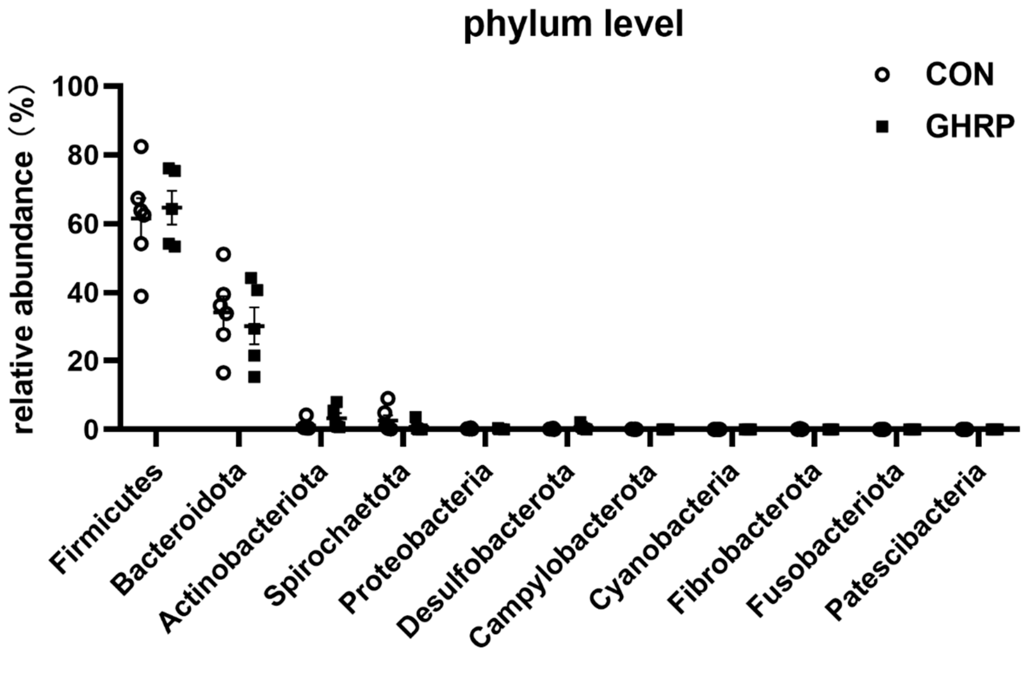

Figure S2. Relative abundance of phyla in the colon of pigs. Values are means ± SEMs; n = 6. CON, Injection of saline; GHRP, Injection of [D-Lys3]-GHRP- 6.
